# Empowering Patient Autonomy: The Role of Large Language Models (LLMs) in Scaffolding Informed Consent in Medical Practice

**DOI:** 10.1111/bioe.70030

**Published:** 2025-09-11

**Authors:** Jemima Allen, Neil Levy, Dominic Wilkinson

**Affiliations:** Monash University; University of Oxford

**Keywords:** Artificial intelligence ethics, informed consent, large language models, medical decision-making, patient autonomy

## Abstract

The principle of (respect for) patient autonomy has traditionally emphasized independence in medical decision-making, reflecting a broader commitment to epistemic individualism. However, recent philosophical work has challenged this view, suggesting that autonomous decisions are inherently dependent on epistemic and social supports. Wilkinson and Levy’s “scaffolded model” of autonomy demonstrates how our everyday decisions rely on distributed cognition and various forms of epistemic scaffolding – from consulting others to using technological aids like maps or calculators.

This paper explores how Large Language Models (LLMs) could operationalize scaffolded autonomy in medical informed consent. We argue that rather than undermining patient autonomy, appropriately designed LLM systems could enhance it by providing flexible, personalized support for information processing and value clarification. Drawing on examples from clinical practice, we examine how LLMs might serve as cognitive scaffolds in three key areas: enhancing information accessibility and comprehension, supporting value clarification, and facilitating culturally appropriate decision-making processes.

However, implementing LLMs in consent procedures raises important challenges regarding epistemic responsibility, authenticity of choice, and the maintenance of appropriate human oversight. We analyze these challenges through the lens of scaffolded autonomy, arguing that successful implementation requires moving beyond simple questions of information provision to consider how technological systems can support genuinely autonomous decision-making. The paper concludes by proposing practical guidelines for LLM implementation while highlighting broader philosophical questions about the nature of autonomous choice in technologically mediated environments.

## I Introduction

Imagine buying a house. You wouldn’t simply hand over your savings based on a brief conversation with the estate agent. You’d likely consult property websites, talk with family members who’ve bought homes before, and definitely seek legal advice. Yet, when it comes to medical decisions, the formal consent process typically centres on patients making independent choices based primarily on a single conversation with their doctor. This disparity, potentially reflects a misunderstanding in how people actually make decisions.^1^ Wilkinson and Levy have recently argued that our decision-making is inherently interdependent, relying on “epistemic scaffolding” – the support structures that enable us to understand and apply our values to complex choices.

In this paper, we extend Wilkinson and Levy’s analysis by examining how artificial intelligence (AI), specifically large language models (LLMs), might serve as novel forms of epistemic scaffolding in medical decision-making. While concerns about AI in healthcare often focus on automation and replacement of human judgment,^2^ we propose a different framework: viewing LLMs as tools that could enhance rather than diminish human autonomy. Specifically, we propose a structured integration of LLMs in informed consent, functioning to bridge the gap between merely providing information and actively supporting the complex cognitive processes required for autonomous medical decision-making.

Medical ethics has been shaped by a distinctly Western, individualistic conception of autonomy that emphasizes independence in decision-making.^3^ The informed consent process emerged as the primary mechanism for operationalizing respect for autonomy. Those that hold individualistic accounts emphasize an agent’s capacity to reflect on and identify with their motivations, acting on desires that one reflectively endorses.^4^ From this perspective, LLMs might appear problematic, potentially inserting a layer of abstraction between the patient’s authentic self and their medical decisions, and subtly shaping or directing patient choices.

However, these individualistic conceptions face several important challenges. Feminist and relational theorists argue that such views of autonomy are overly “atomistic”, with Wolf noting that liberal individualism “strips away relationships that are morally central”.^5^ These writers emphasize how our capacity for self-governance is inherently shaped by and dependent upon social context and consultations with family members, trusted advisors, and healthcare providers.^6^ Rather than seeing autonomy as freedom from influence, relational accounts recognize that autonomous decision-making emerges from supportive social relationships.

The model of “scaffolded autonomy” proposed by Wilkinson and Levy goes beyond relational accounts by recognizing our “epistemic dependence” on others in decision-making to help us understand and apply our values to complex choices.^7^ Just as physical scaffolding provides necessary support for construction, epistemic and social scaffolding promotes autonomous decision-making by supporting understanding, deliberation, and value-application. Research in cognitive science indicates the adaptive outsourcing of understanding, with people often rating their own understanding of a concept as higher when they believe experts understand it. Crucially, this confidence only applies when individuals believe they have access to that expert knowledge.^8^ Wilkinson and Levy claim that these scaffolds aren’t merely helpful additions to an otherwise independent process, but rather actively shape our cognitive processes and are therefore constitutive of genuine autonomy itself. Even those who conceptualize autonomy more individualistically can recognize the practical value of supports that enhance understanding and deliberation.

Healthcare systems already recognize this relational dimension to some extent, with patients consulting family members, specialists, spiritual caregivers, and sometimes ethicists when making significant choices. Yet, current consent processes, shaped largely by legal requirements and traditional interpretations of autonomy, may not always fully support a collaborative approach to decision-making.^9^ They often overemphasize information provision and documentation at the expense of comprehension and value clarification. Meanwhile, family involvement, though providing valuable support, may create ethical tensions from power imbalances and risks of coercion. A scaffolded model should acknowledge these existing tensions, seeking to integrate support while enhancing rather than undermining patient agency.

LLMs offer an opportunity to reconsider how we operationalize respect for autonomy in medical practice.^10^ These tools could transform traditional consent processes by providing more personalized information delivery, supporting ongoing iterative questioning, and improve patients’ understanding (Box 1). Through the structured integration of LLMs in informed consent, patients can engage in sophisticated dialogue about personalized medical information.^11^ However, deploying LLMs as autonomy scaffolds raises novel philosophical and practical questions about ensuring genuinely support rather than subtle manipulation, with implications for doctor-patient relationships, and interactions with other forms of epistemic scaffolding (like family support networks).^12^

In Section II, we explore how LLMs might serve as scaffolding tools, analyzing both their potential benefits and the epistemic responsibilities. In Section III, we address potential objections, including both epistemic and autonomy-related concerns. Section IV concludes by synthesizing the philosophical implications, while providing practical recommendations for implementation.

## II LLMs as Scaffolding Tools for Informed Consent

At its most basic level, LLMs can enhance patients’ access to and understanding of accurate medical information required for informed consent.^13^ LLMs could help standardize the core information provided during consent conversations, potentially reducing the marked variability in risk disclosure that exists even within individual surgeons’ practices,^14^ while still allowing for necessary personalization by adjusting explanations in real-time based on patient understanding and questions. Multiple language support ensures patients can engage with complex medical information in their preferred language,^15^ while 24/7 availability means patients can explore information at their own pace and in the comfort of their own homes.^16^ Through interactive dialogue capabilities, these systems can also engage patients in active discussion about their medical choices, helping them explore options and implications in depth.^17^

It is worth noting that existing consent practices already incorporate various forms of scaffolding, from written materials to discussions with multiple providers. LLMs would not be replacing a completely unscaffolded process, but rather enhancing and extending the scaffolding elements that already exist in best practices for informed consent.

There are concrete ways LLMs might enhance informed consent that should be recognized even by those who do not embrace our theoretical framework. For example, they could help patients better understand statistical risk information, visualize potential treatment outcomes, or systematically explore how different options align with their priorities. These practical benefits remain valuable whether one views such supports as constitutive elements of autonomy itself or as helpful tools that facilitate an otherwise independent decision-making process.

LLMs could provide digital transcripts of consent conversations, including the exact questions and concerns raised by patients and the LLM’s detailed explanation in response. LLMs could then automatically summarize and analyze these transcripts to create *consent interaction reports* for clinicians to review later. These reports could include not only a summary of the information provided and the patients’ key concerns, but also identify any red flags in the consent conversation indicating (either explicitly or implicitly) that the patient lacks understanding, voluntariness or capacity for informed consent. Such comprehensive digital records might offer more detailed insights into the consent process than traditional clinical documentation, creating an audit trail that could improve legal documentation and provide valuable feedback for improving consent processes.

However, LLMs could support patient autonomy in ways that go beyond simple information transfer. Drawing on Wilkinson & Levy’s work on scaffolding autonomy, LLMs could be used to help scaffold informed consent in several ways.

Firstly, LLMs could be designed to help patients “give precision” to their values in medical contexts.^18^ Wilkinson and Levy argue that patients need help making their abstract values more precise and applicable to specific situations.^19^ Through iterative dialogue, an LLM could help patients to not only clarify and articulate their values in medical contexts, but also to understand how different treatment options might align with or challenge these articulated values.

Secondly, an LLM suitably designed for scaffolding consent could support the distributed nature of a patient’s medical decision-making. Rather than treating decisions as isolated patient choices, LLMs could facilitate the complex network of relationships and conversations through which medical decisions typically emerge. The importance of these conversations is already recognized in current practice – for complex decisions, healthcare providers often encourage family involvement and provide resources for shared decision-making. However, LLMs could potentially enhance and systematize these existing supports. This could involve helping patients effectively share and explain medical information with family members, friends, and other trusted advisors by converting technical explanations into more accessible language or generating discussion guides for family conversations. Furthermore, LLMs could help patients integrate insights from multiple sources – whether from different healthcare providers, family members’ experiences, or cultural and religious perspectives – helping them identify areas of consensus and conflict while preparing questions to resolve uncertainties.

LLMS can also help to overcome problems arising from the fact that patients may refrain from asking multiple questions or worry about wasting doctors’ time with self-described “trivial queries” in relation to consent.^20^ This self-censoring behaviour can significantly impact the quality of informed consent, as patients may leave consultations with unaddressed concerns or incomplete understanding. LLMs could serve as low-stakes intermediaries, allowing patients to explore concerns and questions without time pressure or social anxiety.

Beyond translating into different languages, LLMs can also facilitate communication by adapting communication styles to support different cultural frameworks for medical decision-making. While Western medicine theoretically emphasizes individual patient autonomy, research shows that in practice, Western patients, like those from other cultures, typically make medical decisions through consultation and collaboration with family members, friends, and trusted advisors. As Levy argues, the apparent difference between Western ‘individualistic’ approaches and Asian ‘collective’ decision-making may reflect differences more in theoretical frameworks than in actual practice – we are individualists in theory but not in practice.^21^ This suggests that scaffolded consent processes through LLMs aren’t just appropriate for ‘collective’ cultures but reflect how people actually make decisions across cultural contexts.

That said, there are still important cultural variations in how this shared decision-making is expressed and structured institutionally. Many Asian healthcare systems explicitly recognize family involvement and collective decision-making,^22^ sometimes formally incorporating community leaders’ input,^23^ or showing stronger institutional deference to medical authority.^24^ Some cultures employ high-context indirect communication about serious illness, using euphemisms or non-disclosure,^25^ in contrast to Western medicine’s typically more direct approach. The challenge lies not in choosing between individual or family-based decision-making, but in creating consent processes that effectively integrate familial epistemic scaffolding while safeguarding against inappropriate influence – a challenge that applies across cultural contexts but takes culturally specific forms. LLMs could be programmed to navigate these cultural nuances by adjusting how options are presented, incorporating culturally-appropriate discussion frameworks, using suitable metaphors and explanations, and modifying the directness of communication while still ensuring patient autonomy – all while recognizing that the need for decision-making support and scaffolding is universal rather than culture-specific.

Any such system would need robust safeguards against cultural stereotyping and oversimplification of diverse cultural practices, while still maintaining sufficient structure to be practically useful in clinical settings. The optimal approach might involve a combination of system-level defaults based on regional and demographic patterns, alongside both explicit preference settings and adaptive responses to each patient’s actual cognitive and decision-making style as revealed through their interactions. This dual tracking of stated preferences and observed patterns recognizes that there may be significant variation between how people think they ought to make decisions and how they actually process information and arrive at choices most effectively.

LLMs might avoid such stereotyping by allowing patients to specify their explicit preferences, such as their desired level of direct communication about diagnoses,^i^ preferred involvement of family members, or specific cultural practices that affect their healthcare decisions. Departures from the culturally appropriate communication style might serve as a default, to be adjusted by explicit patient preference or by LLMs adapting to each patient’s demonstrated cognitive patterns and decision-making approach – for instance, noting whether they process information better through concrete examples versus abstract explanations, prefer to think through scenarios step-by-step or holistically, or engage more effectively with numerical versus narrative presentations of risk, regardless of their stated cultural preferences or background. This recognition of intra-cultural variation and individual cognitive styles aligns with Levy’s observation that our actual decision-making practices often differ from our theoretical commitments about how we ought to make decisions.^26^

Over time, the LLM could learn from each patient’s interactions, building a nuanced profile that reflects both their explicit preferences and their revealed cognitive and decision-making patterns. Furthermore, there would need to be clear protocols for situations where cultural preferences might conflict with clinical best practices or legal requirements.

## III Challenges & Limitations

While LLMs offer promising possibilities for scaffolding informed consent, their implementation raises significant challenges that warrant careful consideration. We will address these challenges under two headings: first, the epistemic challenges that LLMs may pose, and second, the possibility that they might undermine, rather than enhance, patient autonomy. In addition, there are equally significant practical challenges to implementing such systems in clinical settings. These include technological reliability concerns (ensuring consistent, accurate information delivery), social trust and adoption issues (patients’ willingness to engage with AI systems for sensitive medical decisions), digital literacy and access barriers (ensuring equitable benefit across diverse patient populations and age groups), and institutional integration challenges (incorporating LLMs into existing healthcare IT systems and workflows). Some of these practical implementation issues have been addressed elsewhere.^27^ However, in this paper, we focus primarily on the philosophical and ethical dimensions of LLM-mediated consent, examining how these systems might reshape the cognitive architecture of medical decision-making and patients’ capacity for authentic choice.

### Epistemic Challenges

To understand how LLMs might support or undermine autonomous decision-making, we must first examine how these systems might integrate with and extend our cognitive processes. These include establishing appropriate trust and verification mechanisms, ensuring information quality and accuracy, and maintaining effective integration with expert medical knowledge. However, how we conceptualize these challenges depends crucially on our understanding of trust and verification in cognitive processes.

Record and Miller argue that responsible use of sophisticated technologies requires active checking practices that might seem incompatible with the kind of automatic trust needed for cognitive extension.^28^ However, this may represent an overly stringent interpretation of what automatic trust requires. Clark and Chalmers’ view is more nuanced – they suggest that technological extensions can be part of the mind when they achieve a default level of trust comparable to biological processes.^29^

Consider how we interact with our biological cognitive processes: we don’t consciously verify every memory or sensory input, but we maintain background processes that automatically flag potential inconsistencies or errors. When recalling events, our minds automatically cross-reference details against other memories to check for coherence. When seeing objects, our visual system automatically compensates for lighting conditions and perspective. These verification processes operate as a form of background epistemic vigilance without disrupting our default trust in these cognitive systems.

Similarly, skilled users of technological systems often develop comparable background verification processes that operate without disrupting the primary task. Just as we automatically notice when a memory seems fuzzy or a visual perception seems odd, experienced users develop an automatic sense of when technological outputs might need verification. This isn’t active distrust, but rather a form of integrated epistemic vigilance that becomes part of the fluent use of the technology.

Applied to LLMs in medical consent, this suggests that appropriate verification mechanisms need not preclude the kind of default trust required for genuine cognitive integration. Physicians might automatically scan LLM explanations for alignment with their understanding of patient values, much as they unconsciously monitor their own language choices when explaining procedures. Patients might develop fluid ways of triangulating LLM information against their prior medical knowledge and their physician’s guidance, just as they naturally cross-reference new medical information against their lived experience.

This understanding suggests that the key epistemic challenge isn’t how to balance trust against verification, but rather how to design LLM interactions that support the development of appropriate background verification processes. These include ensuring that all LLM interactions are clearly documented and accessible to both the patient and healthcare team, creating a transparent record that supports automatic cross-referencing. The medical information provided by the LLM should also undergo regular validation by clinical experts to ensure accuracy and alignment with current guidelines, with clear processes for updating content as medical knowledge evolves – similar to how our biological knowledge systems are constantly updated through experience and learning.

Human oversight should be maintained through integration with existing clinical workflows – the physician’s initial discussion of values and preferences guides the LLM interaction, while their final verification of consent ensures appropriate understanding has been achieved. Additionally, clear protocols must be established for identifying and correcting any errors or misunderstandings that arise during the LLM interaction, with established pathways for escalation to human healthcare providers when needed

This reconceptualization of epistemic challenges helps clarify how LLMs might be genuinely integrated into the consent process while maintaining appropriate safeguards. However, as we will see in the following section, these epistemic considerations interact with broader autonomy-related challenges that must also be carefully addressed.

### Autonomy-Related Challenges

The primary concerns about the use of LLMs in informed consent relate to their potential to undermine rather than enhance patient autonomy. These challenges can be organised into three broad subcategories: impacts on decision-making capacity, threats to authentic decision-making, and relational concerns.

The first category concerns how LLMs might inhibit patients’ internal psychological reasoning processes by introducing additional layers of complexity to the consent process, and thereby impeding their ability to make decisions. Beyond deciding about medical procedures themselves, integrating LLMs into the consent process means that patients must now navigate choices about whether or how they wish to engage with the technological system – a meta-decision that risks adding to their cognitive workload and inhibiting their decision-making capacity. This complexity is compounded by the paradoxical nature of digital interaction: while LLMs offer convenient access to information and guidance, this very convenience may reduce meaningful engagement with the consent process. Just as we observe with digital terms and condition agreements where users habitually “click through” without careful consideration;^30^ patients might treat LLM-mediated consent processes as mere procedural hurdles, rather than opportunities for genuine understanding and deliberation.^31^ This could be addressed through carefully designed interfaces that chunk information appropriately and use progressive disclosure techniques and comprehension checks to maintain engagement.

Further complicating these concerns is the fundamental limitation of LLMs in detecting and responding to human emotional states.^32^ Unlike human clinicians who can recognize non-verbal cues, assess psychological capacity, and identify signs of distress or confusion, text-based LLMs (currently) lack this crucial ability to recognize when patients might need additional support or intervention. In the medium term, integration of multimodal interaction capabilities (i.e. systems with audio-visual functions that can interpret facial expressions and body language cues)^33^ and systems for flagging emotional or psychological concerns for the treating physician to review could help bridge this gap. But in the nearer term, without these additional programming features, this technological ‘blindness’ to human emotional states could result in missed opportunities for necessary human intervention in the consent process, potentially compromising patient care and autonomy if not recognized and addressed. Clear escalation pathways to human providers should be established for cases where emotional support or complex decision-making assistance is needed.

Additionally, there is the risk that LLMs, even when designed to help patients give precision to their values, might inadvertently interfere with this formative process by subtly steering patients toward certain value hierarchies or by resolving ambiguities prematurely. For instance, an LLM that guides a patient through value clarification exercises might, through its framing questions or sequential organization, implicitly suggest certain trade-off rates between competing values like longevity versus quality of life. This creates a tension: while LLMs could enhance patient understanding of complex medical information, they might simultaneously diminish opportunities for patients to exercise “formative autonomy”^34^ in settling their own values. This involves not just choosing options based on pre-existing preferences, but rather forming or constituting one’s preferences through the very act of choosing. Addressing this tension requires designing LLM systems that support reflective deliberation without imposing value hierarchies, perhaps by explicitly presenting multiple frameworks for weighing competing values, encouraging patients to articulate their own reasoning processes, and creating space for preference formation rather than merely eliciting preference.

The second category of autonomy-related challenges addresses how LLMs might fail to engage effectively with patients’ authentic values or preferences. A significant concern is that patients might defer uncritically to LLM guidance, viewing artificially intelligent systems as more authoritative or objective than their own judgment. This “automation bias” – the tendency to give undue weight to computer-generated recommendations – has been well-documented in other healthcare contexts and could potentially undermine patient autonomy in LLM-mediated consent processes.^35^ Unlike human doctors, who can actively encourage patients to voice their own views and concerns, LLMs might inadvertently create a dynamic where patients feel less inclined to question or challenge the information presented, viewing it as more definitive or authoritative than intended or warranted. However, it is important not to idealize the patient-physician relationship: it’s unclear to what extent patients are actually more inclined to voice their concerns in consent conversations with human doctors. On the contrary, it may be that in certain clinical circumstances patients feel more comfortable raising their concerns with an LLM than with a human doctor, viewing LLM-mediated interactions as “private and anonymous”.^36^ One consideration for future empirical evaluation is that the use of LLMs as scaffolds in informed consent might increase the number of patients who refuse treatment (e.g., because they don’t feel any pressure or social risk in declining to proceed during a virtual interaction). As an additional consideration, LLMs should be designed with explicit transparency about their role as support tools rather than authority figures in the consent process, regularly prompting patients to reflect on their own values and preferences rather than simply accepting system-generated recommendations.

A further consideration in LLM-mediated consent involves how these systems might help balance valuable family involvement with patient autonomy. The scaffolded autonomy model recognizes that family input often enhances decision quality while acknowledging that family dynamics can sometimes undermine patient agency. LLMs could be specifically designed to navigate this tension by: (1) giving patients granular control over what medical information is shared with family members; (2) providing patients with language and frameworks to effectively incorporate family input while maintaining their decision-making authority; (3) helping patients distinguish between constructive family support and inappropriate pressure; and (4) creating documentation that clearly establishes the patient’s considered preferences even when these differ from family recommendations. This approach recognizes family as a potential epistemic resource while preserving the ethical principle that competent patients should remain the final authority regarding their medical care.

This ties into broader considerations about the boundary between supportive scaffolding and subtle manipulation in LLM-mediated consent processes. While LLMs can provide valuable support by helping patients organize their thoughts, present information in digestible chunks, and systematically explore their values, they will inevitably influence decision-making through their presentation of information.

Another consideration is how commercial interests might influence the design of LLM-mediated consent processes. Without proper safeguards, LLMs might subtly direct patients toward more lucrative procedures or treatments favored by corporate stakeholders rather than those best aligned with patient values and interests. Since LLMs are trained on vast datasets that may include commercially biased medical literature, these biases could arise even if commercial interests do not intentionally introduce them.

However, it is also important to recognize that human physician decision-making is far from completely free from external influences, including pharmaceutical marketing, institutional pressures to increase procedure volumes, or insurance reimbursement structures that incentivize certain treatment pathways. Studies have demonstrated that even small gifts from industry can affect prescribing patterns,^37^ and fee-for-service payment models may incentivize more interventions regardless of patient benefit.^38^ These human susceptibilities to influence are often implicit, difficult to track, and inconsistently disclosed to patients. Additionally, physicians often fail to recognize the impact of implicit biases on their therapeutic decisions.

As Thaler and Sunstein argue regarding choice architecture generally, such influence is unavoidable^39^ – information must always be presented in some order and framed in some way, whether by human physicians or AI systems. The relevant question is not whether influence exists, but how it compares to existing practices and how transparently it can be examined and optimized.

LLMs offer a potential advantage here: their decision-making processes can be systematically audited, documented, and regulated in ways that individual clinician decision-making cannot be. While human cognition remains largely opaque, algorithmic recommendations can be traced, analyzed for patterns of bias, and corrected when problematic. The biases present in their training data can be systematically analyzed, documented, and adjusted in ways that individual physicians’ implicit biases often cannot be.^40^ For instance, we can audit how LLMs present survival versus mortality statistics, examine patterns in their follow-up questions, or analyze variations in language across different treatment options. This transparency could allow for more rigorous optimization of information presentation than is possible with human providers, whose framing effects and response patterns may be less consistent and harder to study systematically (as well as much more challenging to modify).

Addressing these concerns requires robust governance including mandatory disclosure of commercial relationships, independent auditing for biased recommendations, and separation between LLM developers’ financial interests and healthcare institutions. Patients should be explicitly informed about who developed the system, what commercial relationships exist, and what safeguards protect their care decisions.

The key task for healthcare institutions implementing LLM-mediated consent would be developing robust oversight mechanisms that recognize and optimize these inevitable framing effects. Regular systematic audits would need to examine how information is presented across different patient populations, allowing for identification of any problematic patterns while maintaining clear documentation of default presentation orders and framing choices. Healthcare systems would need to articulate clear rationales for their chosen information structures, offering an improvement on current consent processes which lack standardized communication protocols for transparency and accountability.^41^ Such governance frameworks should include mandatory disclosure of commercial relationships, independent auditing for biased recommendations, and clear separation between LLM developers’ financial interests and healthcare institutions.

These systems would need to be coupled with ongoing assessment of patient outcomes and understanding, creating feedback loops that allow for continuous optimization of presentation patterns. Importantly, all of these processes should regularly benchmark against human physician communication patterns, using existing best practices in patient communication as a practical starting point while leveraging the unique capabilities of LLMs to potentially improve upon them.

It would also be important to ensure that patients understand the nature and limitations of their interaction with an AI system, the potential algorithmic biases (without undermining patient trust), and that they’re engaging with technology rather than a human clinician.

Fundamental ethical questions about AI and transparency would need to be addressed before LLMs are implemented in consent processes. These include how to ensure LLMs maintain neutrality when presenting medical options and how should we address the LLM’s own training biases to prevent them from affecting medical decisions. These concerns raise fundamental questions about algorithmic transparency and ethical implementation in the context of medical decision-making. A key challenge lies in developing reliable mechanisms to detect whether LLMs might be inadvertently steering patients toward particular choices. One possible approach would be to compare decision patterns between LLM-scaffolded and traditional unscaffolded consent processes. If systematic differences emerge in treatment choices between these two groups, this might seem to indicate problematic steering by the LLM system.

However, this comparative approach presents a complex methodological challenge. Any observed differences in decision patterns between scaffolded and un-scaffolded choices cannot straightforwardly be interpreted as evidence of problematic steering. Indeed, if the scaffolding is working as intended (i.e., helping patients better understand their options and more effectively align choices with their values), we would expect to see systematic differences in decision patterns (Box 2).

These differences could reflect enhanced rather than compromised decision-making. The critical question, therefore, is not whether LLM scaffolding influences decisions, but whether it does so in ways that support rather than undermine authentic choice.

This suggests the need for more sophisticated evaluation frameworks that look beyond simple outcome differences to examine the quality and authenticity of the decision-making process itself (Box 3).

Regular auditing using these metrics, combined with qualitative analysis of decision-making patterns, could help distinguish between beneficial scaffolding and problematic steering. The goal is not to eliminate influence entirely – which would be neither possible nor desirable – but rather to ensure that any influence serves to enhance rather than undermine patient autonomy. Additional technical safeguards could be implemented to monitor for potential steering mechanisms (Box 4).

These measures should be combined with regular human oversight and periodic ethical review to ensure that the LLM system maintains its role as a supportive scaffold rather than becoming an undue influence on patient choice.

The third category of challenge concerns how LLMs might affect the relational aspects of medical decision-making. There is the fundamental doctor-patient relationship, which traditionally builds on sustained personal interaction, trust, and mutual understanding developed over time.^42^ This relationship enables doctors to present information in ways tailored to each patient’s needs, while creating an environment where patients feel comfortable expressing concerns and asking questions. The introduction of LLM-mediated consent processes will necessarily alter these relationships, though perhaps not in straightforwardly negative ways.

While LLM-mediated consent might reduce the overall quantity of face-to-face time between physicians and patients, it could paradoxically enhance the quality of their interactions. By delegating the more routine aspects of information provision – explaining basic procedures, listing standard risks, and outlining alternative treatments – to LLMs, physicians could be freed to focus on more meaningful aspects of the consent process. Rather than spending limited consultation time walking through standardized information, physicians might have more opportunity to engage in deeper discussions about patients’ values, detect subtle signs of unwarranted influence, and address specific concerns that emerge from the patient’s LLM interaction. This redistribution of communicative labour could enable physicians to better fulfil their core ethical obligations: ensuring that consent reflects the patient’s authentic values and preferences rather than external pressures or misunderstandings.

However, this potential benefit must be carefully balanced against the risk of technological estrangement. The introduction of LLM-mediated processes, despite their efficiency benefits, could create a sense of technological distance that might affect the development of trust and rapport between physicians and patients. One crucial element of the consent process is to enable physicians to understand not just their patients’ explicit choices, but also their underlying values, fears, and motivations. Therefore, any implementation of LLM-mediated consent must be designed to enhance rather than replace the essential human elements of medical decision-making.

A related concern might be raised about what we might call ‘autonomy atrophy’ – the possibility that reliance on LLM support could gradually diminish both physicians’ and patients’ native capabilities in the consent process. Just as GPS navigation might weaken natural way-finding abilities, extensive reliance on LLMs for medical consent might reduce physicians’ confidence in evaluating consent without technological support, or patients’ abilities to process medical information independently.

This concern mirrors a much older debate about technological augmentation of human capabilities. In Plato’s *Phaedrus*, Socrates argues against the technology of writing itself, claiming it would lead to the atrophy of memory and genuine understanding. He warns that writing would create “forgetfulness in the minds of those who learn to use it, because they will not practice their memory. Their trust in writing, produced by external characters which are no part of themselves, will discourage the use of their own memory within them”.^43^ Yet, history has thoroughly demonstrated that the benefits of writing as an external memory system far outweigh any reduction in individual memorial capabilities. The technology of writing hasn’t weakened human thinking – rather, it has dramatically expanded our collective cognitive capabilities by allowing us to build on and engage with recorded knowledge in ways that would be impossible through memory alone.

Similarly, while LLM assistance might indeed lead to some reduction in physicians’ and patients’ abilities to communicate complex medical information without technological support – such as explaining technical procedures in accessible language, systematically working through risks and benefits, or structuring information in easily digestible ways – this may be more than compensated for by the enhanced ability to support better overall decision-making when the technology is available. The key question is not whether these specific capabilities might diminish, but whether the integrated system of human plus technological support enables better outcomes. Research already shows that healthcare professionals generally lack proper consent training and supervision.^44^ If LLMs can help create more thorough understanding, better value-aligned choices, and more authentic consent processes, then some reduction in our ability to perform these tasks without technological assistance may be an acceptable trade-off for improved supported decision-making.

Moreover, concerns about potential impacts on physician capabilities remain empirical questions requiring investigation. Rather than assuming skill degradation, we need research examining how technological support affects physicians’ capabilities over time. It’s possible that LLM assistance could actually enhance physicians’ abilities by providing consistent models of good consent practices, or by freeing up cognitive resources to focus on more nuanced aspects of patient interaction. The key research questions include: How does LLM integration affect physicians’ development of consent-related skills? Does technological support enhance or diminish physicians’ ability to recognize subtle forms of coercion or undue influence? How might LLM assistance impact physicians’ confidence and competence in handling ethically complex consent scenarios? Future studies should examine these outcomes as LLMs are implemented in clinical settings.

That said, it seems likely that physicians will be required to maintain some basic competencies without technological support. The goal should be to develop implementation frameworks that leverage LLM capabilities while maintaining core human skills – much as we continue to teach basic arithmetic despite the ubiquity of calculators, or basic navigation despite GPS availability. Indeed, the introduction of LLM-based practices might create opportunities to strengthen physician training in consent processes. By automating routine aspects of information delivery, resources and time could be reallocated toward improving the currently inadequate system of consent education for healthcare professionals. This suggests the need for thoughtful integration that not only enhances consent processes through technology but potentially enables better professional development in this crucial area of clinical practice.

## IV Conclusion

The integration of large language models into informed consent processes represents more than just a technological innovation – it invites us to fundamentally reconsider how we understand autonomous decision-making in technologically mediated environments. Our analysis reveals that LLMs could serve as powerful tools for operationalizing scaffolded autonomy, while simultaneously raising profound questions about the nature of autonomous choice in an age of artificial intelligence.

LLMs offer an opportunity to better align theory and practice by explicitly supporting the distributed nature of medical decision-making. Their ability to provide adaptive information delivery while accommodating different decision-making styles could help bridge the gap between theoretical autonomy and practical decision-making processes.

Successful implementation requires moving beyond simplistic concerns about technological influence toward developing frameworks that integrate LLM support with existing human oversight. This means designing systems that enhance rather than replace natural cognitive scaffolding, while maintaining clear processes for physician involvement and adapting to different healthcare contexts and patient populations.

The theoretical insights outlined in this paper may translate to specific practical steps for healthcare institutions (Box 5).

Of course, such approaches face practical limitations. Not all healthcare contexts allow for extended deliberation, and some patients may lack access to or comfort with technological tools. Distinguishing helpful scaffolding from inappropriate influence remains challenging. How can we ensure LLM systems enhance rather than replace patients’ natural cognitive scaffolding? When should technological support defer to human interaction?

The path forward requires careful attention to three parallel streams: empirical evaluation of outcomes, philosophical examination of technologically-mediated autonomy, and practical refinement of implementation strategies. Through this integrated approach, LLM-mediated consent processes could help patients better engage with medical decisions in ways that reflect both their values and their natural decision-making processes.

## Box 2: Expected decision patterns indicating effective LLM scaffolding.

- Greater consistency between stated values and treatment choices
- More frequent selection of complex treatment options that require detailed understanding
- Increased willingness to consider alternative approaches
- Higher rates of questioning initial recommendations

## Footnotes

1 Wilkinson, D., & Levy, N. (2024). Scaffolding informed consent. *Journal of Medical Ethics*. 10.1136/jme-2024-110105

2 Alves, M., Seringa, J., Silvestre, T., & Magalhães, T. (2024). Use of Artificial Intelligence tools in supporting decision-making in hospital management. *BMC Health Services Research, 24*(1), 1282. 10.1186/s12913-024-11602-y

3 Young, R. (1986). Limiting Autonomy: Harm to Self. *Personal Autonomy: Beyond Negative and Positive Liberty. New York: St. Martins*. Beauchamp, T. L., & Childress, J. F. (2019). *Principles of biomedical ethics* (Eighth ed.). New York: Oxford University Press.

4 Dworkin, G. (1988). *The theory and practice of autonomy* (Vol. 20): Cambridge University Press; Frankfurt, H. (2018). Freedom of the Will and the Concept of a Person. In *Agency And Responsiblity* (pp. 77-91): Routledge. Young, R. (1982). The value of autonomy. *The Philosophical Quarterly (1950-), 32*(126), 35-44.

5 Wolf, S. M. (1996). *Introduction: Gender and Feminism in Bioethics*: Oxford University Press, p. 17. Stoljar, N. (2011). Informed consent and relational conceptions of autonomy. *Journal of Medicine and Philosophy, 36*(4), 375-384; Mackenzie, C. (2021). Relational equality and the debate between externalist and internalist theories of relational autonomy. In *Autonomy and Equality* (pp. 32-56): Routledge.

6 Hardwig, J. (1990). What about the family? *Hastings Cent Rep, 20*(2), 5-10. Kukla, R. (2005). Conscientious Autonomy: Displacing Decisions in Health Care. *Hastings Center Report, 35*(2), 34-44. https://doi.org/10.1353/hcr.2005.0025. Fan, R. (2015). Informed Consent: Why Family-Oriented? In R. Fan (Ed.), *Family-Oriented Informed Consent: East Asian and American Perspectives* (pp. 3-23). Cham: Springer International Publishing. Osuji, P. I. (2018). Relational autonomy in informed consent (RAIC) as an ethics of care approach to the concept of informed consent. *Medicine, Health Care and Philosophy, 21*(1), 101-111. 10.1007/s11019-017-9789-7.

7 Wilkinson & Levy, *op. cit*. note 1.

8 Sloman, S. A., & Rabb, N. (2016). Your Understanding Is My Understanding:Evidence for a Community of Knowledge. *Psychological Science, 27*(11), 1451-1460. 10.1177/0956797616662271. Rabb, N., Fernbach, P. M., & Sloman, S. A. (2019). Individual Representation in a Community of Knowledge. *Trends in Cognitive Sciences, 23*(10), 891-902. https://doi.org/10.1016/j.tics.2019.07.011.

9 Ubel, P. A., Scherr, K. A., & Fagerlin, A. (2017). Empowerment failure: how shortcomings in physician communication unwittingly undermine patient autonomy. *The American Journal of Bioethics, 17*(11), 31-39.

10 Aydin, F., Yildirim, Ö. T., Aydin, A. H., Murat, B., & Basaran, C. H. (2023). Comparison of artificial intelligence-assisted informed consent obtained before coronary angiography with the conventional method: Medical competence and ethical assessment. *DIGITAL HEALTH, 9*, 20552076231218141. 10.1177/2055207623121814; Shi, W., Zhuang, Y., Zhu, Y., Iwinski, H., Wattenbarger, M., & Wang, M. D. (2023). *Retrieval-Augmented Large Language Models for Adolescent Idiopathic Scoliosis Patients in Shared Decision-Making*. Paper presented at the Proceedings of the 14th ACM International Conference on Bioinformatics, Computational Biology, and Health Informatics, Houston, TX, USA. https://doi.org/10.1145/3584371.3612956; Xiao, Z., Li, T. W., Karahalios, K., & Sundaram, H. (2023). *Inform the uninformed: Improving Online Informed Consent Reading with an AI-Powered Chatbot*. Paper presented at the 2023 CHI Conference on Human Factors in Computing Systems (CHI ’23), Hamburg, Germany.

11 Ayers, J. W., Poliak, A., Dredze, M., Leas, E. C., Zhu, Z., Kelley, J. B., … Hogarth, M. (2023). Comparing physician and artificial intelligence chatbot responses to patient questions posted to a public social media forum. *JAMA internal medicine*. 10.1001/jamainternmed.2023.1838; Bernstein, I. A., Zhang, Y., Govil, D., Majid, I., Chang, R. T., Sun, Y., … Wang, S. Y. (2023). Comparison of Ophthalmologist and Large Language Model Chatbot Responses to Online Patient Eye Care Questions. *JAMA Network Open, 6*(8), e2330320-e2330320. 10.1001/jamanetworkopen.2023.30320; Ye, C., Zweck, E., Ma, Z., Smith, J., & Katz, S. (2024). Doctor Versus Artificial Intelligence: Patient and Physician Evaluation of Large Language Model Responses to Rheumatology Patient Questions in a Cross-Sectional Study. *Arthritis & Rheumatology, 76*(3), 479-484. https://doi.org/10.1002/art.42737.

12 Allen, J. W., Earp, B. D., Koplin, J., & Wilkinson, D. (2024). Consent-GPT: is it ethical to delegate procedural consent to conversational AI? *J Med Ethics, 50*(2), 77-83. 10.1136/jme-2023-109347.

13 Singhal, K., Azizi, S., Tu, T., Mahdavi, S. S., Wei, J., Chung, H. W., … Natarajan, V. (2023). Large language models encode clinical knowledge. *Nature, 620*(7972), 172-180. 10.1038/s41586-023-06291-2.

14 Aasen, D. M., Wiesen, B. M., Singh, A. B., Piper, C., Harnke, B., Prochazka, A. V., … Meguid, R. A. (2020). Systematic Review of Preoperative Risk Discussion in Practice. *J Surg Educ, 77*(4), 911-920. 10.1016/j.jsurg.2020.02.008.

15 Zhu, W., Liu, H., Dong, Q., Xu, J., Kong, L., Chen, J., … Huang, S. (2023). Multilingual machine translation with large language models: Empirical results and analysis. *arXiv preprint arXiv:2304.04675*.

16 Savage, S. K., LoTempio, J., Smith, E. D., Andrew, E. H., Mas, G., Kahn-Kirby, A. H., … Vilain, E. (2024). Using a chat-based informed consent tool in large-scale genomic research. *Journal of the American Medical Informatics Association, 31*(2), 472-478. 10.1093/jamia/ocad181.

17 Aydin et al., *op. cit*. note 20.

18 Wilkinson & Levy, *op. cit*. note 1.

19 Ibid.

20 Convie, L. J., Carson, E., McCusker, D., McCain, R. S., McKinley, N., Campbell, W. J., … Clarke, M. (2020). The patient and clinician experience of informed consent for surgery: a systematic review of the qualitative evidence. *BMC medical ethics, 21*(1), 58. 10.1186/s12910-020-00501-6.

21 Levy, N. (2023). Too humble for words. *Philosophical Studies, 180*(10), 3141-3160. 10.1007/s11098-023-02031-4.

22 Fan, *op. cit*. note 14, pp. 3-23; Specker Sullivan, L. (2017). Dynamic axes of informed consent in Japan. *Social science & medicine, 174*, 159-168.

23 Resnik, D. B., & Hecking, J. (2020). *Cultural variation in informed consent for clinical research participation*.

24 Lee, S., Lee, W.-h., Kong, B.-H., Kim, I.-S., & Kim, S. (2009). Nurses’ perceptions of informed consent and their related roles in Korea: an exploratory study. *International Journal of Nursing Studies, 46 12*, 1580-1584; Masaki, S., Ishimoto, H., & Asai, A. (2014). Contemporary issues concerning informed consent in Japan based on a review of court decisions and characteristics of Japanese culture. *BMC medical ethics, 15*.

25 Mitchell, J. L. (1998). Cross-cultural issues in the disclosure of cancer. *Cancer practice, 6 3*, 153-160; Paladino, J., Sanders, J. L., Fromme, E. K., Block, S., Jacobsen, J. C., Jackson, V. A., … Mitchell, S. E. (2023). Improving serious illness communication: a qualitative study of clinical culture. *BMC Palliative Care, 22*; Searight, H. R., & Gafford, J. (2005). Cultural diversity at the end of life: issues and guidelines for family physicians. *American family physician, 71 3*, 515-522.

26 Levy, *op. cit*. note 31. ^i^ For example, an LLM could be customised according to the preferred type of physician-patient communication model, as proposed by Zohny, H., Allen, J.W., Wilkinson, D., & Savulescu, J. (2025). Which AI doctor would you like to see? Emulating healthcare provider–patient communication models with GPT-4: proof-of-concept and ethical exploration. *Journal of Medical Ethics* Epub ahead of print: 03 March 2025.

27 Allen, J. W., et al., *op. cit*. note 22.

28 Record, I., & Miller, B. (2018). Taking iPhone Seriously: Epistemic Technologies and the Extended Mind. In J. A. Carter, A. Clark, J. Kallestrup, S. O. Palermos, & D. Pritchard (Eds.), *Extended Epistemology* (pp. 0): Oxford University Press.

29 Clark, A., & Chalmers, D. (1998). The Extended Mind. *Analysis, 58*(1), 7-19.

30 Jones, M. L., Kaufman, E., & Edenberg, E. (2018). AI and the Ethics of Automating Consent. *IEEE Security & Privacy, 16*(3), 64-72. 10.1109/MSP.2018.2701155

31 Allen, J. W., et al., *op. cit*. note 22.

32 Ibid.

33 Driess, D., Xia, F., Sajjadi, M. S., Lynch, C., Chowdhery, A., Ichter, B., … Yu, T. (2023). Palm-e: An embodied multimodal language model. *arXiv preprint arXiv:2303.03378*.

34 Raskoff S. Z. (2022). Nudges and hard choices. Bioethics, 36(9), 948–956. https://doi.org/10.1111/bioe.13091

35 Lyell, D., & Coiera, E. (2016). Automation bias and verification complexity: a systematic review. *Journal of the American Medical Informatics Association, 24*(2), 423-431. 10.1093/jamia/ocw105.

36 van Heerden, A., Ntinga, X., & Vilakazi, K. (2017). *The potential of conversational agents to provide a rapid HIV counseling and testing services*. Paper presented at the 2017 International Conference on the Frontiers and Advances in Data Science (FADS).

37 Fickweiler F, Fickweiler W, Urbach E. (2017). Interactions between physicians and the pharmaceutical industry generally and sales representatives specifically and their association with physicians’ attitudes and prescribing habits: a systematic review. *BMJ Open*, 7:e016408. 10.1136/bmjopen-2017-016408.

38 Gosden, T., Forland, F., Kristiansen, I. S., Sutton, M., Leese, B., Giuffrida, A., Sergison, M., & Pedersen, L. (2000). Capitation, salary, fee-for-service and mixed systems of payment: effects on the behaviour of primary care physicians. *The Cochrane database of systematic reviews*, 2000(3), CD002215. https://doi.org/10.1002/14651858.CD002215.

39 Thaler, R. H., & Sunstein, C. R. (2021). *Nudge: The final edition*: Yale University Press.

40 Ploug, T. (2018). Physicians’ framing and recommendations. Are they nudging? And do they violate the requirements of informed consent? *Journal of Medical Ethics, 44*, 543 - 544.

41 Aasen, D. M., *op. cit*. note 24.

42 Surbakti, E. F., & Sari, K. (2018). The Relationship Between Consultation Length and Patient Satisfaction: A Systematic Review. *KnE Life Sciences, 4*(9). 10.18502/kls.v4i9.3556.

43 Plato. (1925). *Phaedrus* (H. N. Fowler, Trans. Vol. 9). Cambridge, MA: Harvard University Press.

44 Propst, K., O’Sullivan, D. M., Ulrich, A., & Tunitsky-Bitton, E. (2019). Informed consent education in obstetrics and gynecology: a survey study. *Journal of Surgical Education, 76*(4), 1146-1152. 10.1016/j.jsurg.2018.12.005; Smith, N. W. S., Overland, J., & Wagstaff, M. (2016). Informed consent practices by junior medical officers: a cross-sectional study. *ANZ journal of surgery, 86*(1-2), 10-11. 10.1111/ans.13310.

